# Supplementary material for: Metabolic Profiling and Stable Isotope Analysis of Wines: Pilot Study for Cross-Border Authentication
Source: Foods. 2024 Oct 23;13(21):3372. doi: 10.3390/foods13213372 (PMC11545056; doi:10.3390/foods13213372)
Supplement: Supplementary file 1 [file foods-13-03372-s001.zip › foods-3256491-supplementary.pdf]

Supporting Information

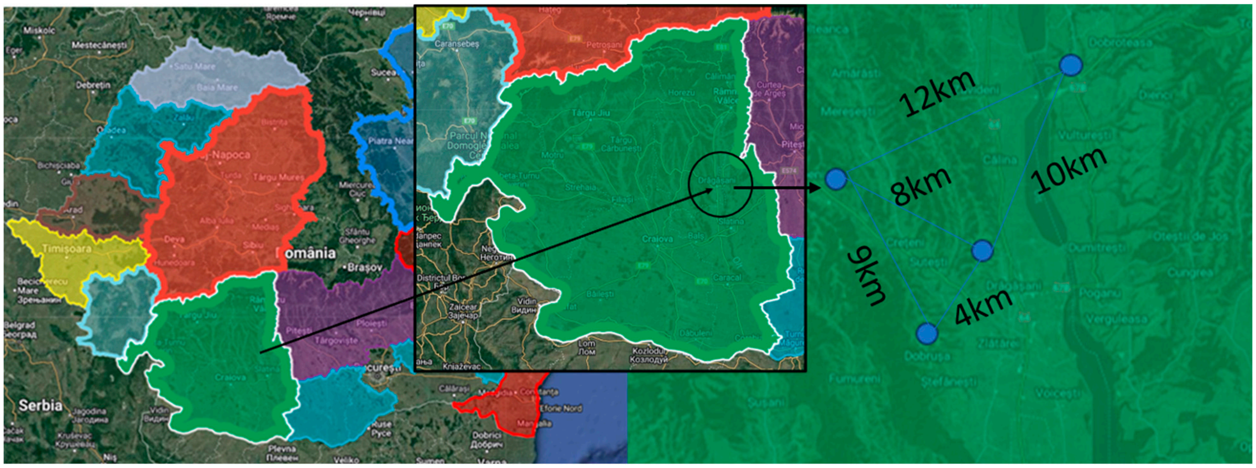

Figure S1. Investigated wine origin source https://www.onvpv.ro/ro/content/indicatii-geografice accessed on 20 September 2024

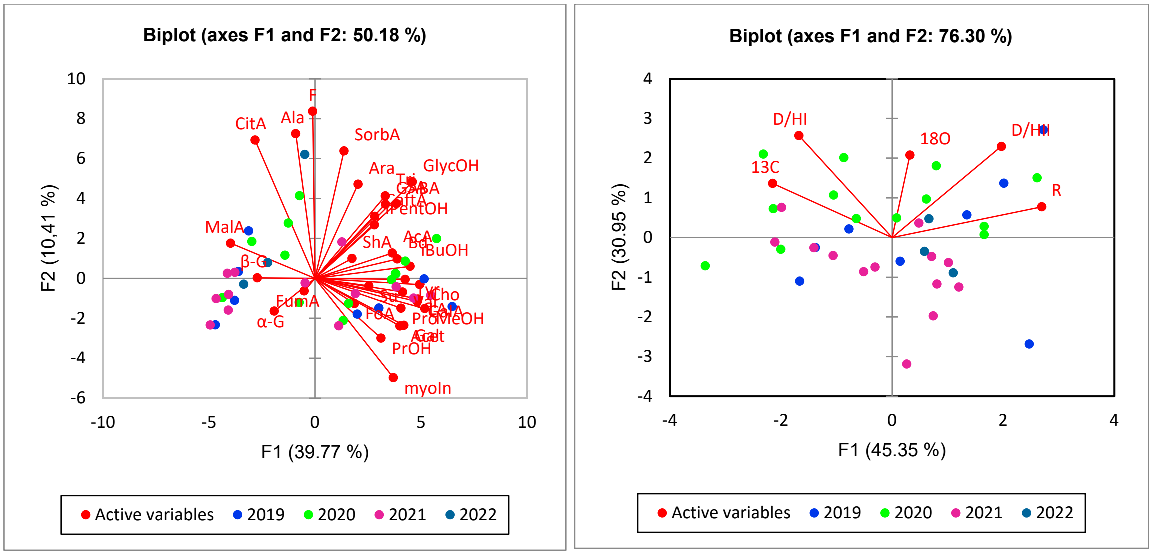

Figure S2. The variables contribution in PCA for the differentiation of wine samples according to harvest year

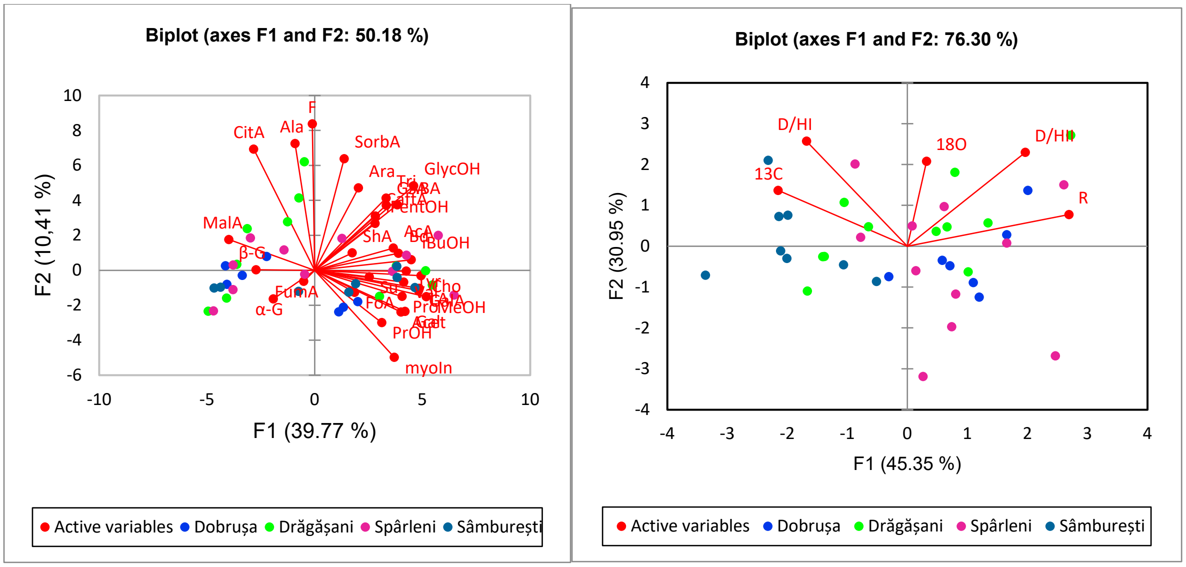

Figure S3. The variables contribution in PCA for the differentiation of wine samples according to geographical origin

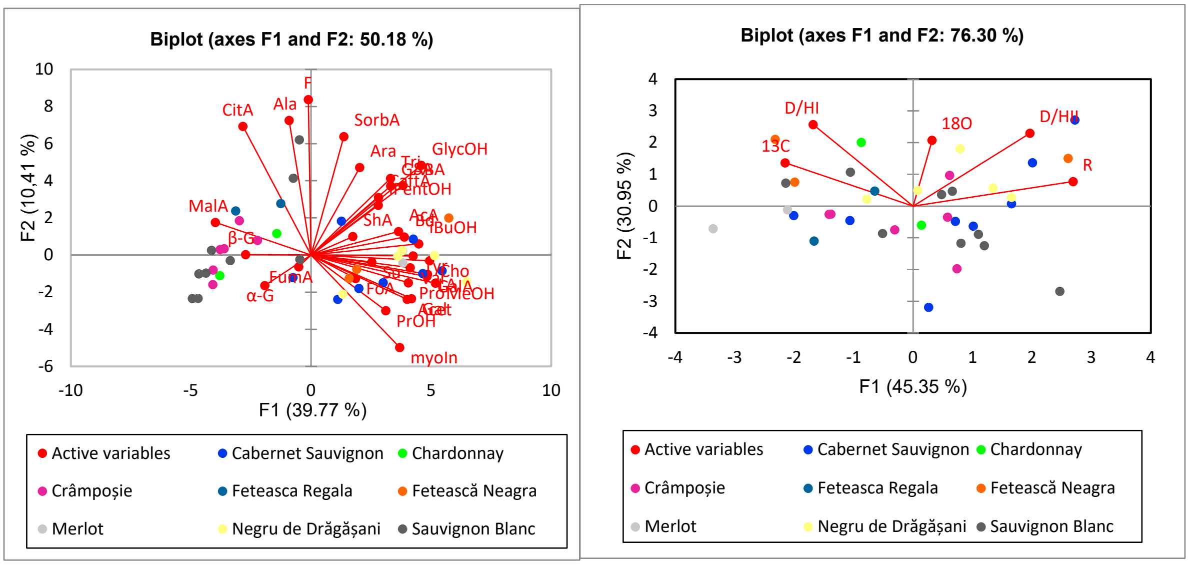

Figure S4. The variables contribution in PCA for the differentiation of wine samples according to grape variety
